# Investigating Causal Relations between Genetic-Related Intermediate Endophenotype and Risk of Chronic Prostatitis: Mendelian Randomization Study

**DOI:** 10.1155/2022/4560609

**Published:** 2022-08-28

**Authors:** Shengfeng Zhang, Xing Xie, Lei Yu, Nili Jiang, Xihuan Wei, Yanling Hu

**Affiliations:** ^1^Department of Biochemistry and Molecular Biology, School of Pre-Clinical Medicine, Guangxi Medical University, Nanning, 53002 Guangxi, China; ^2^Department of Intensive Care Unit, The People's Hospital of Guangxi Zhuang Autonomous Region & Research Center of Intensive Care Unit, Nanning, 530021 Guangxi, China; ^3^Guangxi Academy of Medical Sciences, Nanning, 530021 Guangxi, China

## Abstract

**Objective:**

Prostatitis is a common disease of the male genitourinary system, which seriously disturbs the physical and mental health of male patients. It is related to many factors such as living habits, age, and race, but the etiology has not been fully elucidated. This study investigated whether there is a causal relationship between clinical biochemical indicators (i.e., intermediate phenotype) and prostatitis through Mendelian randomization. The subjects of the study were prostatitis patients and related SNPs in the Guangxi Fangchenggang health examination cohort.

**Methods:**

According to the requirements of Mendelian randomization (MR), the single nucleotide polymorphisms (SNPs) related to prostatitis patients and 29 common SNPs related to clinical biochemical indicators were analyzed by linkage disequilibrium, and the calculated SNPs were selected. Finally, the related SNPs were analyzed by Mendelian randomization method.

**Results:**

15 biochemical indicators such as complement C4, FOL, CRP, HCY, and estradiol have shared chronic prostatitis SNP sites, and five qualified SNPs were finally screened for complement C4. Finally, complement C4 was obtained by Mendelian randomization method (*P* = 0.039), which was statistically significant. The other 28 clinical endophenotypes were all negative.

**Conclusion:**

The results show that there was a causal relationship between complement C4 and prostatitis, and the more consistent SNP is rs2075799.

## 1. Introduction

Prostatitis is the third most common urinary system disease that threatens men's health after benign prostatic hyperplasia and prostate cancer [1]. Overall, it is estimated that 4.5%-9% of the male population is diagnosed with prostatitis, and the recurrence rate is as high as 50% in elderly patients [2]. Prostate symptoms can lead to depression and decreased quality of life [3]. Inflammation has adverse consequences on sperm quality [4] and finally leads to infertility [5], which affects the health of human offspring. This is also the most serious consequence. It is caused by the interaction of various stimulating factors [6]. The causes of its occurrence are varied [7]. Chronic prostatitis (CP)/chronic pelvic pain syndrome (CPPS) is closely related to lifestyle, diet, smoking, gastrointestinal or anorectal diseases, and impaired sexual function [8]. It has even been suggested that high-level spare-time sports activities can reduce the incidence of CP/CPPS. Some people believe that age, race, and geographical area are also important risk factors for chronic prostatitis [9], while others believe that body mass index (BMI) is also a risk factor [10]. The history of moderate to severe lower urinary tract symptoms (LUTS) and prostatic hypertrophy (BPH) is significantly related to prostatitis [11]. Studies have shown that prostatitis-like symptoms are a multifactorial problem closely related to drinking, smoking, frequent sexual intercourse, fatigue, stress, and lack of sleep [7]. Although the results of some observational studies show that lifestyle factors affect CP/CPPS risks, so far, such studies are still few in general [10]. At present, there is no report on the relationship between clinical biochemical indicators and prostatitis. Therefore, our research group conducted linkage disequilibrium (LDSC) analysis on the relationship between prostatitis and clinical in the early stage and found that complement C4 and C3 have significant correlation with prostatitis, but it is not clear whether there is a causal relationship. And Mendelian randomization method is a popular and accurate epidemiological method to study causality. This method can select single nucleotide polymorphisms (SNP) as an instrumental variable, which can avoid the influence of confounding factors such as environmental factors on the relationship between exposure factors and outcomes [12, 13]. Therefore, it is necessary to conduct Mendelian randomization analysis on whether there is a causal relationship between clinical endophenotype and prostatitis.

## 2. Materials and Methods

### 2.1. The Sources of Data

#### 2.1.1. Case Group

The samples were from six large-scale tertiary grade A hospitals in Guangxi. This study received consent and approval from the Medical Ethics Committee of Guangxi Medical University. The diagnosis of CP was carried out according to the CP classification standard of the National Institutes of Health (NIH) [14], and the inclusion and exclusion criteria of samples were established. (1) Inclusion criteria: complaints of long-term and repeated pain or discomfort in the pelvic area, lasting for more than 3 months, may be accompanied by different degrees of urination symptoms and sexual dysfunction, which seriously affects the quality of life of patients; routine examination of prostatitis EPS/semen/urine VB3 bacterial culture after prostate massage was negative. (2) Exclusion criteria: excluding patients with neurogenic bladder, urethral stricture, benign prostatic hyperplasia, prostate cancer, testicular epididymis and spermatic cord diseases, overactive bladder, interstitial cystitis, sexually transmitted diseases, bladder tumors, urinary tuberculosis, stones, and other diseases affecting urination, as well as severe diabetes, cardiovascular diseases, liver and kidney insufficiency, psychosis, habitual diarrhea or inflammatory intestinal diseases, lumbar diseases, central, and peripheral neuropathy, etc.

#### 2.1.2. Control Group

The inclusion criteria were matched by age and sex; normal subjects were selected from unrelated areas in the same area as CP patients. The exclusion criteria were no urinary system-related diseases or tumors and cardiovascular diseases, no diabetes, psychosis, hepatic and renal insufficiency, inertial diarrhea or patients with inflammatory intestinal diseases, lumbar diseases, central, and peripheral neuropathy, etc.

### 2.2. Genome-Wide SNP Genotyping

The first phase of male health samples from the First People's Hospital of Fangchenggang was genotyped on DNA samples using Human Omni 1-Quad chip from Illumina company in the United States. The second phase of male urology outpatient samples from six large-scale tertiary grade A hospitals in Guangxi was genotyped on DNA samples using Human Omni ZhongHua-8 chip from Illumina company in the United States. The brief procedure of the experiment was as follows: amplification of whole genome DNA → endonuclease digestion to fragment DNA → isopropanol precipitation of DNA → DNA resuspension → DNA hybridization with chip → washing → single base extension → staining. After scanning the fluorescence signal by Illumina iScan chip scanning system, the data were obtained according to the different fluorescence emitted by the fluorescent groups represented by different deoxyribose bases. The obtained fluorescence data were analyzed by Genome Studio software to obtain SNP typing data files.

### 2.3. Genotyping Data Filtering

The genotyping data were filtered using PLINK 1.07 software, and strict quality control was performed on the obtained SNPs data, with the following data exclusion criteria: call rate < 0.95, minor allele frequency (MAF) < 0.01, and Hardy-Weinberg equilibrium (HWE) < 1 × 10^−3^.

### 2.4. Genotyping Data Filling

According to the linkage disequilibrium (LD) rules based on Hapmap Phase II Han Chinese in Beijing (CHB) population release#24 panel, the IMPUTE 5 software was to fill the genome of SNP sites that had not been typed, and the sites with a posterior probability greater than 90% were reserved. And based on the same exclusion criteria as above, fill the SNP data after genome filling again.

### 2.5. Selection of Instrumental Variables

In order to better investigate the causal relationship between clinical biochemical indicators and CP, the SNPs we selected need to meet the following criteria: (1) high association between SNPs and clinical biochemical indicators with genome-wide study significance, i.e., *P* < 5 × 10^−8^. (2) SNPs were independent of each other to avoid the bias caused by linkage disequilibrium (LD), when *R*^2^ of LD > 0.01, one of them was eliminated [15].

### 2.6. Evaluation of Instrumental Variables

To ensure that the correlation between instrumental variables and exposure factors was strong, the *F* value of each SNP is usually used to determine the strength of the correlation and to avoid bias from weak instrumental variables, and bias from weak instrumental variables was generally considered to be absent when the *F* value was greater than 10 [16]. The statistical power of the MR analysis of CP was calculated for each SNP using an online tool (http://cnsgenomics.com/shiny/mRnd/). In Mendelian randomization, it was important to ensure that instrumental variables and confounding factors are independent of each other and cannot be indirectly linked to outcome variables through confounding factors. Therefore, SNPs associated with confounding factors were excluded by whether they were associated with confounding factors or not.

### 2.7. Two-Sample Mendelian Randomization

Mendelian randomization estimates the relationship between genetically related intermediate endophenotypes and CP by the following three MR methods: inverse-variance weighted (IVW), MR-Egger regression, and weighted median (WM). IVW is one of the commonly used methods, but it presupposes that all instrumental variables are valid, and as long as one SNP does not satisfy the assumptions of instrumental variables, this method will be biased. Although multiple genetic variants can enhance the statistical power of Mendelian randomization analysis, due to the existence of pleiotropy, the causal relationship with CP is biased when some genetic variants do not satisfy the assumption of instrumental variables [16]. However, when 50% of SNPs are effective instrumental variables, WM can obtain estimates consistent with the final effect [17]. Under the internal assumption that instrumental variables are independent of direct effects, the MR-Egger regression provides a valid effect estimate even if all SNPs are invalid instrumental variables [18].

### 2.8. Sensitivity Analysis and Heterogeneity Test

To further evaluate the effect of heterogeneity on the causal estimation, Cochran's *Q* test was used to evaluate the heterogeneity test of the instrumental variables [19], while one SNP in turn was excluded and the remaining SNPs were continued to be analyzed by the Mendelian randomization method, i.e., leave one out (LOO) for sensitivity analysis of the results [20]. Statistical analysis was performed using the “TwoSampleMR” package in R software (v3.6.1 https://www.r-project.org), and differences were considered statistically significant at *P* < 0.05.

## 3. Results

### 3.1. Screening of Mendelian Randomization Instrumental Variables (Shared SNP Sites)

The overall situation of the respective shared SNP sites associated with 29 clinical biochemical indicators of chronic prostatitis is shown in Table 1; 15 biochemical indicators such as complement C4 and FOL have shared SNP sites with CP, with the number of shared sites ranging from 1286. 14 biochemical indicators such as IgA and BUN have no shared SNP sites with CP. Shared SNP sites of chronic prostatitis and 15 clinical biochemical indicators are shown in Table 1. Chronic prostatitis has a total of 286 shared SNP sites associated with complement C4, and after a chain imbalance analysis, there are five SNP sites that can be used as instrumental variables (as shown in Table 2), with *F* values ranging from two to 12, partially biased by weak instrumental variables.

### 3.2. Estimation Results of Mendelian Randomization Method

**Figure 1:**
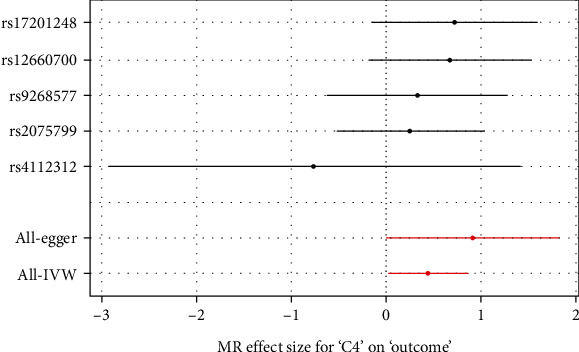
Results of Mendelian randomization method for complement C4 and chronic prostatitis (CP).

Inverse-variance weighted results showed that there was a causal relationship between exposure (complement C4) and chronic prostatitis (OR = 1.040, *P* = 0.039), as shown in Figure 1. The remaining four methods: weighted median estimator (OR = 1.154, 95% CI: 0.192~6.940, *P* = 0.143), Mendelian randomization Egger regression (OR = 1.067, 95% CI: 0.452~2.520, *P* = 0.077), simple mode (OR = 1.209, 95% CI: 0.457~3.201, *P* = 0.193), and weighted mode (OR = 1.212, 95% CI: 0.470~3.122, *P* = 0.165), settled with a *P* value > 0.05 (as shown in Table 3). Among them, 15 clinical biochemical indicators (exposure) and chronic prostatitis (outcome) shared SNPs (after linkage disequilibrium correction) ranging from one to two. Clinical biochemical indicators include complement C4, complement C3, immunoglobulin M (IgM), C-type reactive protein (CRP), alpha-fetoprotein (AFP), ferritin (FERR), vitamin B12, low-density lipoprotein (LDL), high-density lipoprotein (HDL), follicle-stimulating hormone (FSH), estradiol, uric acid, sex hormone-binding globulin (SHBG), FOL (folic acid), and HCY (homocysteine). Using six Mendelian randomization statistical methods, the calculation results of the causal effect of 14 clinical biochemical indicators on CP are negative; that is, there is no causal relationship with CP (outcome), as shown in Table 4.

### 3.3. Sensitivity Analysis

**Figure 2:**
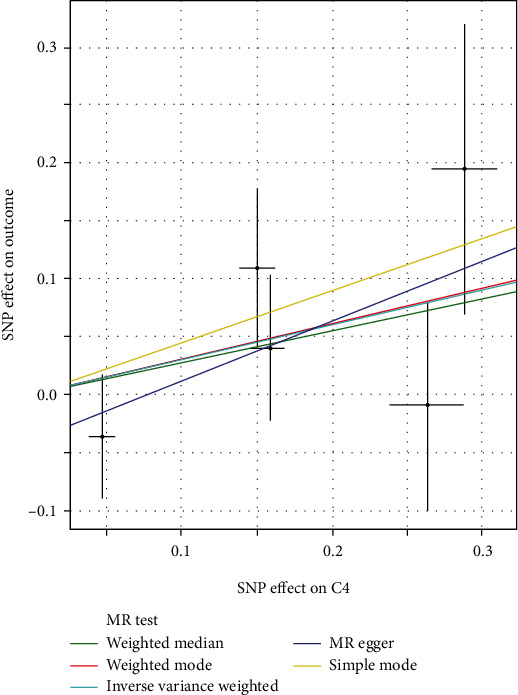
IVW, MR-Egger regression, and WM scatter plot to study the effect of C4 on CP.

**Figure 3:**
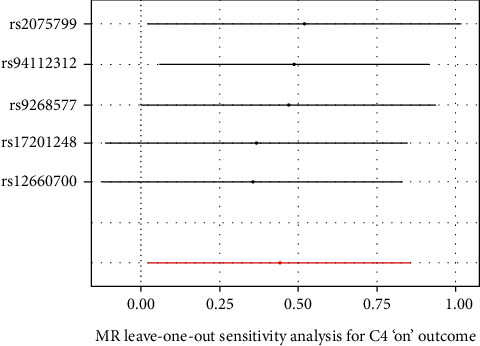
Sensitivity analysis of the leave-one-out method.

To ensure the credibility of this study, the MR-Egger method was used to test the instrumental variables. As shown in Figure 2, genetic pleiotropy does not bias the results. Meanwhile, less heterogeneity among SNPs was observed with the IVW method (*Q* = 0.88, *P* = 0.64). In the sensitivity analysis, we eliminated one SNP in turn and analyzed the remaining SNPs, and there was no one SNP that had a significant effect on the outcome effect, as shown in Figure 3.

## 4. Discussion

Mendelian randomization (MR) is an important epidemiological method that can be used for causal reasoning [21–23]. It uses SNP site data as an instrumental variable to explore the causal relationship between exposure factors and results. Compared with traditional observation and study, it reduces the bias caused by confounding factors and reverse causality and improves its accuracy and scientificity [24]. Compared with randomized controlled trials, it is called a natural randomized control by the epidemiological community. Genetic variation is usually inherited independently, which means that they are usually in a specific relationship [25]. Even if there are unmeasured confusing factors, Mendelian can be used for causal inference [10]. Mendelian randomization studies using biochemical indicators (including inflammatory markers) for chronic prostatitis have not been reported so far. In this study, we used six statistical methods (MR-Egger, weighted median, inverse-variance weighted, simple mode, weighted mode, and Wald ratio) of MR to estimate the causal relationship between 29 commonly used clinical biochemical indicators and prostatitis. Among the 29 biochemical indicators, only 15 clinical indicators met the aforementioned hypothesis of MR, and the remaining 14 did not meet the conditions and were excluded.

Complement C4, complement C3, CRP, and IL-6 are commonly used in clinic to evaluate inflammation, but no causal relationship between C3, CRP, IL-6, and prostatitis was found in this study. However, Hartwig et al. [26] calculated that sIL-6R was positively correlated with the occurrence of schizophrenia through Mendelian randomization method and also pointed out that some effects were mediated by CRP [26]. Our study results show that there was no causal relationship between most clinical indicators and prostatitis. Considering the possibility of weak instrumental variables leading to bias or the presence of genetic pleiotropy that was excluded. Of course, it is also true that many of the 29 indicators included in this study (except procalcitonin, high-sensitivity C-reactive protein, and IL-6) have not been proven to be directly related to prostatitis inflammation in the clinical, meaning that the results of the observational study are consistent with the results of our Mendelian randomization study in this project.

Prospective studies have shown that genetic susceptibility to CRP levels is positively associated with the risk of infection in adults [27, 28]. At present, many literatures have confirmed that complement C4 and C3 are related to inflammation [29, 30]. They are important clinical biochemical markers of the human immune system, and changes in their levels can reflect the state of immunity. Complement C4 and C3 can represent the level of inflammation. For example, the levels of complement C4 and C3 in noncritical and critical patients with covid-19 are different. It is reported that in children with stable asthma, the level of complement C3 was significantly higher than that in the normal control group, and there was no significant difference in the level of complement C4 [31]. It was considered that complement C3 is positively correlated with asthma [31]. It was also considered that the complement C3 of asthmatic children was significantly higher than that of the control group, and there was no significant difference in complement C4 [32]. Studies have confirmed that inflammation and immune factors (IgE, complement C4, complement C3, CRP, ASO, and RF) and hormone elements (Osteoc, FSH, testosterone, and insulin) are significantly related to the occurrence of prostatitis [33]. It showed that this study was highly consistent with the previous research results of Chen et al. [33]. There were five SNP sites with a positive causal association between complement C4 and chronic prostatitis in this study. Among them, s2075799 (*F* = 11.58), rs12660700 (*F* = 9.13), rs17201248 (*F* = 8.61), rs2075799 (*F* = 11.58), rs4112312 (*F* = 2.36), and rs9268577 (*F* = 4.20), only the *F* value of rs2075799 is 11.58. According to the ideal state, the *F* value was greater than 10, there was no weak instrumental variable, while it was consistent with the result resolution consistency of multiple randomization methods. Among them, rs2075799 (*F* = 11.58) was the ideal SNP site that meets the above criteria. The SNP of rs2075799 exist in the MHC II region of 2-mb on the chromosome, and this SNP site is highly correlated with the level of complement C4. rs2075799-related genes are related to schizophrenia, but there is no literature reported that it was related to chronic prostatitis. In MR-Egger analysis of complement C4, its intercept was close to zero, the *P* value was less than 0.005, and there was no horizontal pleiotropy, which was consistent with the results of inverse-variance weighted operation. The statistical results of the two are consistent, which makes it clear that the results are credible. With the development of MR methodology, a multivariate MR Egger regression analysis method for adjusting multieffects has been proposed, which was beyond the range that can be explained by genetically estimated exposure factors and has the same direction, but it was larger and more complex [18]. These studies indicated that complement C4 can be used as a biochemical marker for causal inference of chronic prostatitis. This will be of great significance to the diagnosis, treatment, and prevention of prostatitis. As we all know, acute prostatitis can be diagnosed by comprehensive clinical manifestations, biochemical examination of prostatic fluid, and examinations such as CT. However, the diagnosis is confirmed by prostate finger examination, prostate biopsy, and pathology of surgical resection; these invasive operations will bring great pain and risks to the patient. However, it has been reported that prostatitis is positively correlated with prostate cancer, which is an important high-risk factor for the development of prostate cancer [34]. The potential continuous stimulation of chronic inflammation, the immune status of the prostate, the inflammatory mediators and cytokines of the prostate, and proliferative inflammation and atrophy were high-risk factors for prostate cancer, which suggested that local inflammation of prostate and damage of prostate in systemic inflammatory reaction may lead to the occurrence or progress of prostate cancer [35]. Some also believe that the prostate was cancerous through oxidative stress and reactive oxygen species. Inflammation and atrophic hyperplasia were high-risk factors for prostate cancer, and that inflammation was a possible factor for the generation or development of cancer [36]. Therefore, early intervention of chronic prostatitis will also be of great significance to the prevention of chronic prostatitis [37]. If a noninvasive, accurate, safe, effective, fast, and convenient detection method for early diagnosis can be found, it will bring great benefits to many patients and is also the key to early prevention and treatment of early prostatitis.

From the perspectives of statistics, genetics, and epidemiology, we used a two-sample Mendelian randomization method for the first time to analyze the causal relationship between clinical indicators and CP, which confirmed that there is a causal relationship between complementC4 and CP.

Although the sample size of this research group is not large, the sample comes from the same region and has a certain regional representation, which reduces the bias caused by different populations. The study of Mendelian randomization method is very strict with the standards of exposure factors and causal correlation. There are more Mendelian randomization study methods, each with its own basic conditions, and each method has its own advantages and disadvantages. The application of MR methods must require that any single SNP site must be strongly correlated with exposure factors, not weakly correlated and correlated with confounding factors, and it must be a unidirectional positive relationship, and there must be no multiple effects and reverse causality. It has been suggested that various methods such as sample size calculation and model hypothesis can be used to solve potential methodological problems [32]. There are many determinants of the effect of MR, including the frequency of using genetic variation, the size of the impact of variation on risk factors, the strength of genetic instrument and the size of the study sample, and the strength of the relationship between exposure factors and outcomes [38, 39]. The best way to improve the reliability of the study results is to increase the study sample further.

With the development of genome-wide association studies and the continuous increase of genetic association-related data and the opening of public data, the current application of Mendelian randomization has a basis of data sources and gradually appears to be relatively simple and more widely used. However, it is still difficult to obtain reliable results from database surveys, because in the traditional inverse-variance weighting, all genetic variations must be effective instrumental variables to obtain consistent results. So sensitivity analysis of genetic variation was carried out by median regression method and MR-Egger regression method in Mendelian randomization study [40].

This study is still deficient in that only a few of the 29 clinical endophenotypes have one to two shared SNP sites, especially when there are less than five SNP sites. Except IVW method, other methods should be inconclusive. This may lead to false negatives. This may be due to the relatively small sample size.

However, this is based on the original data provided by the current GWAS study, which is a retrospective study, and the existing statistical data does not increase the number of SNP sites by increasing the number of samples. In the future, when more sample data are added and the relevant SNP sites are expanded, subsequent researchers can conduct MR analysis of these intermediate endophenotypes again.

## 5. Conclusion

In a word, MR can find the causal relationship between exposure factors and genetic variation of outcome from molecular mechanism. The MR study of complement C4 and CP in this study shows that there is a causal relationship between complement C4 and CP, which can provide a new idea and method for the molecular mechanism and immune mechanism of prevention and treatment of chronic prostatitis. If further study can confirm, it is of great significance in the early treatment and prevention of chronic prostatitis and even in the prevention of prostate cancer.

## Figures and Tables

**Table 1:** Clinical biochemical indicator shared SNP sites with chronic prostatitis.

Exposure (indicators)	Outcome (CP) number of shared SNP	Exposure (indicators)	Outcome (CP) number of shared SNP	Exposure (indicators)	Outcome (CP) number of shared SNP
Complement C4	286	FSH	2	IgA	0
FOL	20	IgM	1	BUN	0
CRP	15	LDL	1	IgE	0
HCY	15	FERR	1	Insulin	0
Estradiol	15	SHBG	1	ALT	0
Uric acid	4	TG	0	TE	0
Complement C3	3	Cholesterol	0	Creatinine	0
B12	2	BMI	0	Glucose	0
HDL	2	ASO	0	Triglyceride	0
AFP	2	IgG	0		

Note: CP: chronic prostatitis; FOL: folic acid; CRP: C-type reactive protein; HCY: homocysteine; B12: vitamin B12; HDL: high-density lipoprotein; AFP: alpha-fetoprotein; FSH: follicle-stimulating hormone; LDL: low-density lipoprotein; FERR: ferritin; SHBG: sex hormone-binding globulin; TG: triglyceride; BMI: body mass index; ASO: antistreptolysin O; IgG: immunoglobulin G; IgA: immunoglobulin A; IgE: immunoglobulin E; BUN: blood urea nitrogen; ALT: alanine transaminase; SNP: single-nucleotide polymorphism.

**Table 2:** Exposure (complement C4) and outcome (CP) instrumental variable SNP sites.

SNP ID	SE	*P* value	Exposure	*F*
rs12660700	0.288	0.044	1.51*E* − 38	9.13
rs17201248	0.150	0.164	1.91*E* − 36	8.61
rs2075799	0.159	0.197	2.33*E* − 49	11.58
rs4112312	-0.048	0.350	4.83*E* − 08	2.36
rs9268577	-0.177	0.099	1.19*E* − 16	4.20

Note: SE is standard error.

**Table 3:** Mendelian randomization to estimate the values of causal effect of complement C4 on chronic prostatitis (CP).

Exposure (indicators)	Outcome (CP) shared SNP	Statistical method	*β* value	SE value	*P* value	OR value	CI value
Complement C4	5	MR-Egger	0.915	0.464	0.143	1.154	0.192~6.940
		Weighted median (WM)	0.439	0.248	0.077	1.067	0.452~2.520
		Inverse-variance weighted (IVW)	0.441	0.214	0.039	1.040	0.438~2.468
		Simple mode (SM)	0.497	0.317	0.193	1.209	0.457~3.201
		Weighted mode (WM)	0.483	0.285	0.165	1.212	0.470~3.122

**Table 4:** Mendelian randomization to estimate causal effect values for 14 clinical biochemical indicators of CP.

Exposure (indicators)	Outcome (CP) shared SNP	Statistical method	*β* value	SE value	*P* value
LDL	1	MR-Egger	/	/	/
		Weighted median	/	/	/
		Inverse-variance weighted	/	/	/
		Simple mode	/	/	/
		Weighted mode	/	/	/
		Wald ratio	-0.164	0.249	0.510

SHBG	1	MR-Egger	/	/	/
		Weighted median	/	/	/
		Inverse-variance weighted	/	/	/
		Simple mode	/	/	/
		Weighted mode	/	/	/
		Wald ratio	0.793	0.654	0.225

Uric acid	1	MR-Egger	/	/	/
		Weighted median	/	/	/
		Inverse-variance weighted	/	/	/
		Simple mode	/	/	/
		Weighted mode	/	/	/
		Wald ratio	-0.005	0.003	0.078

Estradiol	2	MR-Egger	/	/	/
		Weighted median	/	/	/
		Inverse-variance weighted	0.093	0.839	0.912
		Simple mode	/	/	/
		Weighted mode	/	/	/
		Wald ratio	/	/	/

FERR	1	MR-Egger	/	/	/
		Weighted median	/	/	/
		Inverse-variance weighted	/	/	/
		Simple mode	/	/	/
		Weighted mode	/	/	/
		Wald ratio	0.000	0.001	0.840

FSH	1	MR-Egger	/	/	/
		Weighted median	/	/	/
		Inverse-variance weighted	/	/	/
		Simple mode	/	/	/
		Weighted mode	/	/	/
		Wald ratio	0.887	0.477	0.063

HDL	1	MR-Egger	/	/	/
		Weighted median	/	/	/
		Inverse-variance weighted	/	/	/
		Simple mode	/	/	/
		Weighted mode	/	/	/
		Wald ratio	-0.869	0.693	0.210

IgM	1	MR-Egger	/	/	/
		Weighted median	/	/	/
		Inverse-variance weighted	/	/	/
		Simple mode	/	/	/
		Weighted mode	/	/	/
		Wald ratio	0.248	0.573	0.665

B12	1	MR-Egger	/	/	/
		Weighted median	/	/	/
		Inverse-variance weighted	/	/	/
		Simple mode	/	/	/
		Weighted mode	/	/	/
		Wald ratio	0.002	0.001	0.164

CRP	1	MR-Egger	/	/	/
		Weighted median	/	/	/
		Inverse-variance weighted	/	/	/
		Simple mode	/	/	/
		Weighted mode	/	/	/
		Wald ratio	-0.102	0.164	0.537

AFP	1	MR-Egger	/	/	/
		Weighted median	/	/	/
		Inverse-variance weighted	/	/	/
		Simple mode	/	/	/
		Weighted mode	/	/	/
		Wald ratio	-0.925	0.634	0.145

Complement C3	1	MR-Egger	/	/	/
		Weighted median	/	/	/
		Inverse-variance weighted	/	/	/
		Simple mode	/	/	/
		Weighted mode	/	/	/
		Wald ratio	-1.389	1.165	0.233

FOL	20	MR-Egger	0.334	0.233	0.168
		Weighted median	0.008	0.027	0.763
		Inverse-variance weighted	0.007	0.021	0.734
		Simple mode	0.010	0.047	0.832
		Weighted mode	0.010	0.043	0.815
		Wald ratio	/	/	/

HCY	15	MR-Egger	0.008	0.022	0.742
		Weighted median	-0.005	0.010	0.635
		Inverse-variance weighted	-0.004	0.008	0.590
		Simple mode	-0.009	0.016	0.591
		Weighted mode	-0.004	0.014	0.749
		Wald ratio	/	/	/

Note: CP: chronic prostatitis; LDL: low-density lipoprotein; FOL: folic acid; CRP: C-type reactive protein; HCY: homocysteine; B12: vitamin B12; HDL: high-density lipoprotein; AFP: alpha-fetoprotein; FSH: follicle-stimulating hormone; FERR: ferritin; SHBG: sex hormone-binding globulin; IgM: immunoglobulin M.

## Data Availability

The labeled dataset used to support the findings of this study is available from the corresponding author upon request.
